# Experimental Chronic Noise Is Related to Elevated Fecal Corticosteroid Metabolites in Lekking Male Greater Sage-Grouse (*Centrocercus urophasianus*)

**DOI:** 10.1371/journal.pone.0050462

**Published:** 2012-11-20

**Authors:** Jessica L. Blickley, Karen R. Word, Alan H. Krakauer, Jennifer L. Phillips, Sarah N. Sells, Conor C. Taff, John C. Wingfield, Gail L. Patricelli

**Affiliations:** 1 Dept of Evolution and Ecology, University of California Davis, Davis, California, United States of America; 2 Dept of Neurobiology, Physiology and Behavior, University of California Davis, Davis, California, United States of America; 3 University of Montana, Wildlife Biology Program, Missoula, Montana, United States of America; University of Milan, Italy

## Abstract

There is increasing evidence that individuals in many species avoid areas exposed to chronic anthropogenic noise, but the impact of noise on those who remain in these habitats is unclear. One potential impact is chronic physiological stress, which can affect disease resistance, survival and reproductive success. Previous studies have found evidence of elevated stress-related hormones (glucocorticoids) in wildlife exposed to human activities, but the impacts of noise alone are difficult to separate from confounding factors. Here we used an experimental playback study to isolate the impacts of noise from industrial activity (natural gas drilling and road noise) on glucocorticoid levels in greater sage-grouse (*Centrocercus urophasianus*), a species of conservation concern. We non-invasively measured immunoreactive corticosterone metabolites from fecal samples (FCMs) of males on both noise-treated and control leks (display grounds) in two breeding seasons. We found strong support for an impact of noise playback on stress levels, with 16.7% higher mean FCM levels in samples from noise leks compared with samples from paired control leks. Taken together with results from a previous study finding declines in male lek attendance in response to noise playbacks, these results suggest that chronic noise pollution can cause greater sage-grouse to avoid otherwise suitable habitat, and can cause elevated stress levels in the birds who remain in noisy areas.

## Introduction

Anthropogenic noise is becoming ubiquitous as natural landscapes are increasingly dominated by humans, but we still have much to learn about the impacts of chronic noise exposure on wildlife [1]–[3]. Recent studies have shown that some species avoid developed areas with high noise levels, reducing available habitat and potentially leading to reduced populations [4]–[6]. However, there is variation among species and individuals in the tendency to avoid noise [4], [5], [7], which raises the question of whether animals that remain suffer detrimental effects, or if these individuals are better able to habituate to noise or are less susceptible to its effects. It has been suggested that animals remaining in (or unable to leave) noisy areas may have lower survival and reproductive success [8]–[10]; indeed, recent studies have demonstrated complex effects of noise on community structure and on breeding and pairing success [4]–[6], [11]. Given the ubiquity of noise in the environment, it is critical that we understand noise impacts on animals whether they remain in or avoid disturbed areas.

One possible impact of introduced noise on animals is the induction of stress, which may be defined broadly as nonspecific adverse effects in vertebrates but is most often characterized by its influence on neuroendocrine physiology. The duration of noise exposure affects the stress response of animals exposed to it [12]. Exposure to a brief but loud noise event, such as a single sonic boom, will result in an acute stress response. An acute stress response is characterized by a rapid release of epinephrine and norepinephrine (the “fight or flight” response) followed by a hypothalamic-pituitary-adrenal (HPA) cascade. The HPA cascade results in increased secretion of glucocorticoid hormones, cortisol or corticosterone, in the blood. Long-term exposure to a chronic noise stressor, such as a high-traffic freeway, can lead to chronic stress, defined as long-term overstimulation of coping mechanisms. This in turn can lead to less predictable changes in the HPA axis. Acclimation or exhaustion may result in reduced glucocorticoid release to the same or novel stressors; facilitation, conversely, can lead to elevated glucocorticoid release in response to novel stressors, and even in cases of reduced peak glucocorticoid response, deficits in negative feedback may develop that result in greater overall exposure to glucocorticoids due to prolonged elevation [12], [13].

Glucocorticoid hormones and their metabolites are commonly used to measure a stress response [14]–[16]. Glucocorticoid hormones can be measured from blood samples or their metabolites may be measured non-invasively from fecal samples as an index of the relative physiological stress of animals [17]–[19]. Glucocorticoid hormones play a major role in allocating energy, and prolonged exposure due to chronic stress can affect fitness by inhibiting resource allocation to reproductive or immune activities, a condition known as allostatic overload [12], [20]–[24].

Studies in captive animals have found that noise can increase HPA activity and glucocorticoid levels [25], [26]; indeed studies of stress physiology often use noise exposure as a method to induce a stress response [27], [28]. Previous observational and experimental studies on the impacts of anthropogenic noise on glucocorticoid levels in wild animals have yielded mixed results. Snowmobile and wheeled-vehicle traffic was associated with elevated fecal glucocorticoid metabolites in wolves and elk [14]. Noise is one potential mechanism of this impact, but visual and other types of disturbance may also contribute to these responses; indeed, the quieter activity of Nordic skiing also correlates with FCMs in capercaillie (*Tetrao urogallus*) [29]. Delaney et al. [30] found behavioral responses in spotted owls to loud noise from visually hidden chainsaws and helicopters, but subsequent studies found no evidence of change in FCMs with exposure to quieter chainsaw noise (below behavioral response threshold) or road proximity to nesting sites [31]. Results from chronic noise studies on humans have also been mixed [32]. Studies of children in areas with high road noise have found increased overnight glucocorticoid levels in urine, as well as impaired circadian rhythms, sleep, memory and concentration, [33] and increased heart-rate responsiveness to acute stressors [34]. However, a study in children living in communities near airports found increases in some measures of stress (blood pressure, epinephrine and norepinephrine) but no similar elevation in overnight urinary cortisol [35]. These results indicate that noise may have a significant effect on glucocorticoids and other stress-related variables in many species, but that further study is needed to determine the degree and extent of these effects and how the effects may vary with different types of noise.

In this study, we test the hypothesis that chronic noise causes an increase in stress levels of lekking greater sage-grouse. We used fecal levels of immunoreactive corticosteroid metabolites (FCMs) as an index of physiological stress and compared FCMs for breeding males on display grounds (leks) with and without experimentally introduced noise. The greater sage-grouse, an iconic species once widespread in western North America, is now declining throughout its range, leading to its listing as an endangered species in Canada and its recent designation as “warranted but precluded” for listing under the Endangered Species Act in the USA [36], [37]. Over the last decade, natural gas development has expanded rapidly across much of the sage-grouse range and has been implicated in reduced lek attendance and abandonment of long-occupied (often for decades) lek sites by males [e.g. 38,39–41]. Males typically gather on lekking grounds for several hours in the early morning when conditions are quiet and still, a time when they may be particularly vulnerable to disturbance from noise pollution from natural gas development and other sources [42]. To investigate whether noise exposure may have contributed to declines in lek attendance, Blickley et al. [43] experimentally introduced noise from natural gas development activities (drilling and road noise) on leks over three breeding seasons (2006–2008). This noise playback caused immediate and sustained declines in sage-grouse lek attendance. Further, different types of noise had different degrees of impact, with drilling noise and road noise causing an average 29% and 73% decline in lek attendance, respectively, compared to their paired controls. That study provides evidence that anthropogenic noise from energy development causes some males to avoid attending leks with introduced noise, but we do not yet know whether noise also has a negative impact on the individuals that remain on noisy leks. The lekking season is a time of high metabolic demand [44] and stress [45] for males, so exposure to noise during this period may have a greater fitness cost.

Here we compare the FCM levels of male sage-grouse on control leks and leks with experimentally introduced noise in the second and third seasons of experimental noise playback (2007 and 2008) [43]. We predict that if noise exposure leads to chronic stress, male sage-grouse on experimental leks will have higher FCMs than males on control leks. Such differences in observed FCM levels may also be observed if males with low glucocorticoid levels are more likely to disperse from noise-treated leks, so we compared the variance in FCM levels on noise and control leks. We also investigated whether elevated FCM levels were associated with declines in peak male attendance on leks to determine the value of this metric as a tool for predicting lek declines.

## Materials and Methods

### Study Area & Experimental Design

**Figure 1:**
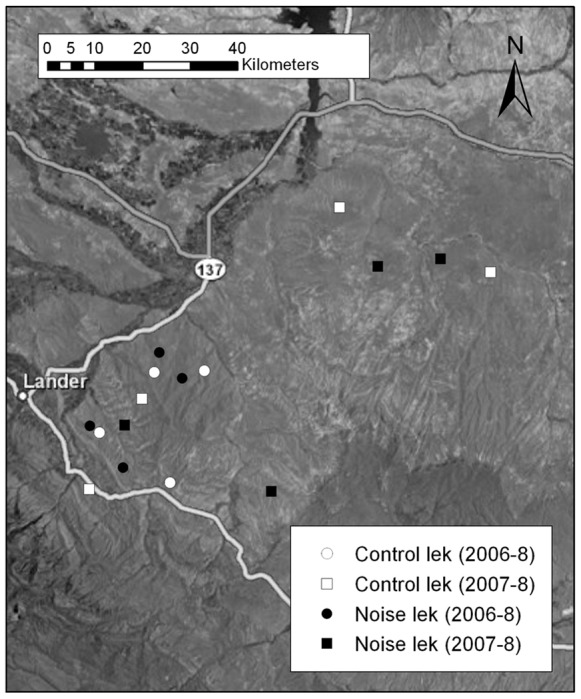
Noise playback study area in Fremont County, Wyoming, USA, 2006–2009. Experimental and control leks were paired on the basis of size and geographic location (the four leks in the upper right are part of the Riverton region, whereas the rest of the leks are in the Lander region).

Study sites were located on federal land relatively undisturbed by human development in Fremont County, Wyoming (42° 50′, 108° 29′30). We monitored a total of 16 leks that were divided into 8 pairs, with the leks of a pair matched according to size and location (6 pairs near the town of Hudson and 2 pairs near the town of Riverton) (Figure 1). Of the 8 lek pairs, 4 pairs were randomly assigned to each noise type, such that there were 4 “drilling pairs”, each including one lek exposed to drilling noise and a similar lek as its control, and 4 “road pairs,” each with one road noise and a matched control. For 3 of the pairs, one lek within a pair was randomly assigned to the treatment (noise) group and the other assigned as control. For the fourth pair, the treatment and control leks were deliberately assigned due to another study that was in progress. During sample collection periods, both leks in a pair were normally visited on the same day.

Noise and playback methods have been previously described [43] and are summarized here. Noise was played beginning in mid-February to early March and continuing through the end of April of each year. Noise was recorded from drilling and main road sites at the Pinedale Anticline natural gas fields and played back using a commercial car amplifier and 3–4 rock-shaped outdoor speakers placed along one edge of the lek. On leks with road-noise playback, recordings of semi-trailer trucks and pickup trucks were combined with 30- and 60-second files of silence at a ratio reflecting the average number of each truck type found on a main energy field access road; these files were then played using the “random shuffle” feature on an MP3 player. Most shift changes occur at 8 am, so our playback may underestimate actual traffic levels during the lekking time. On leks with drilling noise, a 14-minute recording of a drilling rig was played on continuous loop. Natural gas development activities occur 24 hours a day, so noise was broadcast continuously day and night at playback levels that approximate the noise level at 0.25 mile (402 m) from a typical drilling site (JLB and GLP unpublished data). Drilling-noise recordings were broadcast on experimental leks at an equivalent sound level (L_eq_) of 71.4±1.7 dBF (unweighted decibels) SPL re 20 µPa (56.1±0.5 dBA [A-weighted decibels]) as measured at 16 meters; on road-noise leks, where the amplitude of the noise varied with the simulated passing of vehicles, noise was broadcast at an L_max_ (maximum RMS amplitude) of 67.6±2.0 dBF SPL (51.7±0.8 dBA) (see Blickley, et al. [43], for detailed noise-exposure measurements). Noise from playback was localized to each lek due to the small size of our speakers. To control for visual disturbance of the speaker system and researcher presence, control leks had dummy speakers placed in the same arrangement and were also visited to simulate the periodic battery changes on noise leks. This experimental protocol was reviewed and approved by the Animal Care and Use Committee at UC Davis (Protocol # 16435) and the Wyoming Game and Fish Department (Permit # 33–405).

In the first year of the experiment (2006), we played noise on only 4 of the 8 lek pairs (2 experimental leks with introduced drilling noise, 2 with introduced road noise). Therefore, some leks had been exposed to noise the breeding season prior to the first year of FCM measurement; however, we detected no significant impact of duration of noise exposure on lek attendance [43], so years of noise exposure was not included as a potential explanatory variable in candidate model sets.

### Collection of Fecal Samples

Fecal samples were collected from leks soon after all sage-grouse had left the lek for the morning. Samples were collected twice per year from each lek (once during the mid season [April 4–6 in 2007, April 6–8 in 2008] and once during the late season [April 23–26 in 2007, April 22–24 in 2008]) and were collected from paired leks on the same day. Samples were collected using a sweep-search method in which the entire lek was systematically searched and fresh fecal samples were collected individually in Whirl-Pak bags and labeled with a location on the lek relative to the speakers (or dummy speakers). To minimize the chance of collecting multiple fecal samples from the same individual, we collected samples that were a minimum of 5 meters apart, roughly the minimum territory size of a male sage-grouse. Jankowski [45] found lower FCM levels in female sage-grouse than in breeding male sage-grouse. Therefore to avoid collecting samples from females, we collected samples on dates when female visitation is rare; if there were more than 1–2 females on the lek on a potential collection day, sampling for that lek pair was postponed until the next day. Time to collect samples varied among leks from 20–80 minutes. Samples were frozen at −20°C within a few hours of collection until processing. Jankowski et al. [45] found no difference in FCM levels for greater sage-grouse samples held for variable times up to 16 hours prior to freezing.

### Extraction & Radioimmunoassay of Cort

We used extraction and assay procedures, with minor modifications, that were previously validated for application to greater sage-grouse by Jankowski et al. [46]. Individual fecal pellets were kept on ice while uric acid (often present in a discrete cap on the pellet) was removed and discarded. Samples were then lyophilized and returned to storage at −20°C. On the day of extraction, individual fecal pellets were weighed to the nearest 0.0001 g, then manually homogenized, vortexed, and shaken in 5 mL of 80% methanol for at least 30 minutes. Longer incubation in methanol often occurred due to the large number of tubes in each assay, but experimentation with overnight extraction produced no substantial change in detected metabolites. Samples were centrifuged at 5000 rpm for 30 minutes, then 1.5mL of supernatant was drawn off, placed in a separate tube, dried under streaming air in a 70°C water bath and reconstituted in 1.0 mL of steroid diluent provided in the RIA kit (see below). For some very large samples, it was not possible to remove 1.5 mL; in these cases, 500 µL of supernatant was drawn off and reconstitution volume was adjusted accordingly after drying. Extracts were covered with Parafilm and stored at 4°C until assayed.

A pooled sample was made by homogenizing a collection of multiple samples from one control lek (Monument lek) in a blender prior to lyophilization. From this pooled sample, 0.5 g was assayed initially to determine parallelism with the RIA standard curve, and one or more pooled samples were included in each extraction and assay.

Radioimmunoassays were conducted according to the manufacturer's instructions (catalog # 07-120103, MP Biomedicals, Costa Mesa, CA) using 1∶16 dilution of reconstituted extract. This RIA kit utilizes a rabbit-produced BSA IgG polyclonal antibody against corticosterone-3-carboxymethyloxime. This antibody has been widely used for fecal assays due to its ability to bind a broad spectrum of corticosteroid metabolites [47]. Samples were randomly distributed among assays with respect to year and treatment to minimize any impacts of inter-assay variation.

FCM measures were adjusted for the mass of the fecal sample (ng ICM/g sample) to account for differences among leks in fecal pellet mass. In dividing ICM by sample mass, we effectively assume that the relationship between sample mass and fecal transit time (during which corticosteroid metabolites are secreted into the lumen of the gut) is positive and linear. To guard against faults in this assumption, we ran the same statistical analyses using “per sample” FCM data and found no difference in the main effects as reported.

### Statistical Analysis

Fecal glucocorticoid metabolites levels were natural log-transformed to meet assumptions of normality and homoscedasticity prior to analysis. We used an information theoretic approach to evaluate the support for alternative candidate models using Akaike's Information Criterion for small sample sizes (AIC_c_) [48]. Candidate models for the overall effect of noise (Noise effect models) were linear mixed-effect models that assessed the relationship between explanatory variables and the concentration of FCMs collected from experimental and control leks. Potential explanatory variables included pair type (NoiseType, drilling or road noise), control status (Treatment, noise or control), pellet/collection distance from speakers (SpeakerDist), maximum lek size for that year (MaxSize), location (Hudson or Riverton), season (early or late April), and relevant interactions (see Table 1 for full set of candidate models). All models contained lek pair ID, and year (2007 or 2008) as random effects.

**Table 1:** Mixed-effect candidate models for the effect of noise playback on mass-dependent FCM concentrations (natural log-transformed).

Modela,b	*K* c	ΔAIC*_c_* d	*w_i_* e
Treatment^f^	5	0	0.66
Treatment + Location	6	2.4	0.20
Treatment + Location + Treatment:Location	7	4.7	0.06
Null- random effects only	4	5.5	0.04
Treatment + Season	6	6.5	0.03
Treatment + Season + Treatment:Season	7	10.0	<0.01
Treatment + NoiseType + Treatment:NoiseType	7	10.8	<0.01
Treatment + Location + NoiseType + Treatment:Location + Treatment:NoiseType	9	11.2	<0.01
Treatment + NoiseType + Season + Treatment:Season + Treatment:NoiseType	9	20.7	<0.01
Treatment + MaxSize + Treatment:MaxSize	7	25.3	<0.01
Treatment + NoiseType + Season + Treatment:NoiseType + Treatment:Season + Treatment:NoiseType:Season	11	27.3	<0.01
Treatment + SpeakerDistance + Treatment:SpeakerDistance	7	27.5	<0.01
Treatment + NoiseType + MaxSize + Treatment:NoiseType + Treatment:MaxSize	10	35.4	<0.01
Treatment + NoiseType + SpeakerDistance + Treatment:NoiseType + Treatment:SpeakerDistance	9	38.2	<0.01
Treatment + NoiseType + MaxSize + Treatment:NoiseType + Treatment:MaxSize + Treatment:NoiseType:MaxSize	12	45.1	<0.01
Treatment + NoiseType + SpeakerDistance + Treatment:NoiseType + Treatment:SpeakerDistance + Treatment:NoiseType:SpeakerDistance	11	60.4	<0.01

a Abbreviations of predictor variables in methods.

b All models contain lek pairing and year as a random effect.

c Number of parameters in the model.

d Difference in AIC*_c_* (Akaike's Information criteria for small sample size) values from the top ranking model.

e Akaike weight (Probability that the model is the best fit model giving the data and model candidate set).

f Model with substantial support (ΔAIC*_c_* <2).

We also evaluated a set of candidate models that assessed the relationship between the concentration of FCMs on experimental leks and the declines in peak male attendance from the previous year (attendance models). Models contained lek ID and year (2007 or 2008) as random effects. Models were ranked on the basis of differences in AICc scores (ΔAICc) and were assigned Akaike weights (w_i_) corresponding to the degree of support. We calculated model-averaged coefficients and variable importance (sum of variable weights for all models in which the variable was included) for variables contained in all models that received strong support (ΔAICc <2). We also compared the variance in FCM concentrations measured on noise and control leks using a Levene's test. All statistical analyses were performed in R (version 2.12.1, R Development Team 2010).

## Results

We measured baseline fecal immunoreactive corticosterone metabolites of 103.2 and 119.9 ng/g for control and treatment groups, respectively (Table 2). These values are lower than baseline measures of approximately 149 ng/g obtained previously for breeding male greater sage-grouse in Nevada, from which fecal samples were collected after capture [45].

**Table 2:** Parameter estimates (± SE) and relative variable importance for variables in highly supported models (ΔAIC*_c_* <3).

Variable	Parameter estimates^a^	Parameter estimates (back-transformed)^b^	Relative variable importance^c^
Intercept	4.63 (.06)	103.2^d^	-
Treatment:Noise	.15 (.04)	16.7^d^	0.96
Location: Hudson	0.02(.01)	2.9^d^	0.26

a Parameter estimates are natural-log transformed.

b SE not included due to back-transformation.

c Relative variable importance is the summed total of the model weights for models containing that variable.

d Intercept value was added to parameter estimates prior to back-transformation and then subtracted.

**Figure 2:**
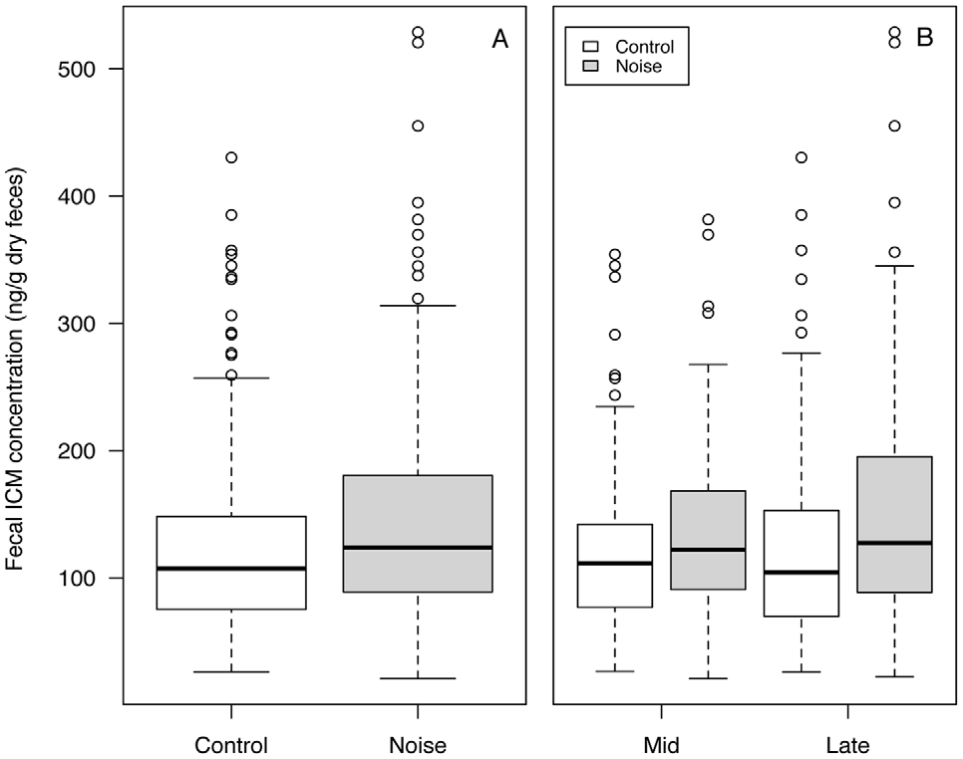
FCM concentrations from control and noise-treated groups. Data shown (A) pooled by season and (B) for mid and late season samples. Horizontal line represents the median value, box ends represent upper and lower quartiles, whiskers represent maximum and minimum values and open circles represent outliers. Plots present measured FCM values, not model output, which is presented in Table 2.

Males on leks exposed to noise had higher (16.7% on average) FCM levels compared with controls (w_i_ = 0.96, Table 1, 2; Figure 2). While models that included the effect of Treatment (noise versus control) were highly supported by the data, there was little support for an interaction of Treatment with NoiseType variable (w_i_ = 0.01, Table 1), indicating that while noise exposure was associated with increased cort, there was little difference in FCM levels between leks with drilling versus road-noise playback. Candidate models containing other possible explanatory variables, including distance from the nearest speaker (SpeakerDist), maximum size of the lek (MaxSize), the regional location of the lek in the Hudson area or Riverton area (Location) and time of the season (Season), received little support relative to the null model (Table 1, Figure 2B), indicating that none of these factors had a strong influence on FCM levels.

To determine whether noise-playback leks with a higher stress response were associated with larger declines in lek attendance, we compared candidate models for the relationship between FCM level and change in lek attendance from the previous year. Only the null model received support (Table 3), indicating that fecal FCM level was not associated with the magnitude of changes in lek attendance on noise leks.

**Table 3:** Mixed-effect candidate models assessing the relationship of FCM concentrations and changes in lek attendance from the previous year on noise-playback leks.

Modela,b	*K* c	ΔAIC*_c_* d	*w_i_* e
Null- random effects only^f^	5	0	0.90
Fecal cort	6	4.6	0.10

a Abbreviations of predictor variables in methods.

b All models contain lek pairing and year as a random effect.

c Number of parameters in the model.

d Difference in AIC*_c_* (Akaike's Information criteria for small sample size) values from the top ranking model.

e Akaike weight.

f Model with substantial support (ΔAIC*_c_* <3).

Finally, we examined whether there was a difference in variance among samples on noise leks and control leks. We found no significant differences in variance between treatment types in 2007 (variance on noise leks = 7729.94, control leks = 6168.28, Levene's W = 0.6327, p = 0.427). Variance on noise leks was significantly higher than on control leks in 2008 (variance on noise leks = 4462.28, control leks = 2758.69, Levene's W = 6.6064, p = 0.01).

## Discussion

We found higher (16.7%) FCM levels on noise-treated leks compared to controls, supporting the hypothesis that chronic noise pollution increases stress levels in male greater sage-grouse. Combined with results from monitoring of lek attendance in the same experiment [43], these results suggest that noise from natural gas development activities can dramatically decrease male attendance on leks and cause physiological impacts on males that remain on noisy leks. The mean level of FCMs in remaining birds was not a good predictor of the degree of decline in peak male attendance on a lek compared with the previous year, indicating that the FCM level measured on a lek is not diagnostic of an effect of noise on peak male attendance (Table 3). Further, we did not find support for an effect of distance from the speakers on FCM levels. Male sage-grouse typically maintain a fixed territory on a lek throughout the season. Within a noise-treated lek, each individual's exposure to noise varied, depending on the location of their territory relative to the speakers. Since noise levels decline exponentially with distance from the speakers, the lack of a distance effect suggests that stress is not exclusively dependent on the noise exposure of individuals. Instead, noise impacted FCM levels on a lek-wide basis.

Blickley et al. [43] found a decline in lek attendance on road-noise leks more than twofold larger than the decline in lek attendance on drilling-noise leks, yet we found no difference in FCM levels between noise-playback types (Table 1, Figure 1). Both noise sources have most of their sound energy ≤2 kHz, but road noise is less predictable than drilling noise and more intermittent, leading to a lower average noise exposure across road-noise leks (43.2±0.89 dBA L_eq_) than drilling-noise leks (56.1±0.45 dBA L_eq_) [43]. Studies on physiological stress in rodents indicate that stressors administered at unpredictable intervals result in greater elevations in plasma corticosterone [49]. Since cort levels may also be implicated in decisions to escape from deleterious conditions [50], we cannot say with certainty that noise type has no differential impact on FCM levels, only that there was no difference observed among males that chose to remain. If road noise did result in a greater cort response in some birds, but the most susceptible birds were also the most likely to disperse, differences would not necessarily be expected among remaining birds. In this scenario, it is likely that variance would be reduced in leks with high losses, reflecting disappearance of individuals with higher FCM levels. Levene's tests did not identify any such difference in variance (indeed, there was a significant difference in one year of the study, but in the opposite direction to predictions). However, the possibility that dispersal is linked to FCM levels cannot be ruled out. Regardless of whether the stress levels of birds on noise leks increased, or whether only high-stress-level individuals remained on noisy leks, these results indicate that chronic noise at leks creates less desirable habitat for greater sage-grouse.

The unknown status of dispersed grouse – and their unknown destinations – leaves several other possible scenarios that should be considered. It is possible that the individuals most likely to disperse could have had different cort profiles at the outset compared with those more prone to remain. If noise playback caused individuals with lower integrated cort to disperse away from noisy leks, that coupled with the possible addition of those birds to control leks could cause trends similar to those observed here. Two possible sources of variation in pre-experiment cort levels among individuals are age and social status [51]–[53]. Reduced juvenile recruitment may have contributed to the observed declines in lek attendance on noise leks, potentially leading to a difference in age structure on noise and control leks [43]; however, this is unlikely to explain the results of this study. Studies of altricial and semi-altricial birds have found lower stress responsiveness shortly after hatching, but responses resemble those of adults by the age of fledging or first molt [54]–[57]. Since young male sage-grouse attending leks are likely to be at least 10 months old and after their first molt, it is unlikely that they would have lower stress response than adults. Social status can also be related to corticosteroid levels [58], therefore social upheaval caused by dispersal between noise and control leks may have contributed to observed FCM levels. Further studies are needed determine whether age-class- and social-status-dependent dispersal in response to noise contributed to the observed results.

Unlike noise sources in most energy development sites, our noise introduction in this study was localized to the immediate lek area, so birds were exposed to noise for only a few hours a day, and only during the breeding season. Therefore, we cannot quantify the effects of noise on FCMs for wintering, nesting or foraging males. Noise at energy development sites is less seasonal and more widespread and may thus affect birds at all life stages, with a potentially greater impact on stress levels. In addition, we looked only at male stress levels in this study, but males and females may respond differently to stress. For example, Jankowski et al. [45] measured FCM levels in sage-grouse in habitats with and without cattle grazing; they found no difference in male FCM levels in response to grazing regime, however, breeding females showed elevated stress response in grazed areas. This suggests that females may be more vulnerable to some types of disturbance; further studies are needed to assess whether female stress levels are influenced by noise.

### Why might noise be stressful?

Increased adrenocortical activity occurs in response to circumstances perceived as threatening by an animal. Although we cannot determine from this study the extent to which noise itself is a threat to sage-grouse, noise may affect social dynamics and increase the perception of threat. Noise may have social impacts on sage-grouse by masking acoustic communication on the lekking grounds [42]. Masking occurs when the perception of a sound is decreased by the presence of background noise, which may reduce the efficacy of acoustic communication. Acoustic signals play an important role in many social interactions, including mate attraction and assessment, territorial interactions, recognition of conspecifics and alarm calling in response to environmental threats [9], [10], [59]. Masking of these acoustic signals may alter or interfere with social interactions and mate choice behaviors [60], [61].

For prey species such as sage-grouse, noise may also increase stress levels by masking the sounds of approaching predators and increasing the perception of risk from predation [62], [63]. The degree to which noise directly affects mortality through changes in predation is largely unknown, as few studies have compared predation rates or hunting success in noisy and quiet areas while controlling for other confounding factors. Francis et al. [4] did so and found that nest predation rates in some songbirds decline in noise-impacted areas, as the dominant nest predator avoided noise. This suggests that noise may cause complicated changes in predator-prey dynamics. Noise may also cause stress due to short-term disruptions in behavior, such as startling or frightening animals away from food or other resources [2], [64]. Further, if individuals associate a particular type of noise, such as road noise, with a danger, such as vehicular traffic, this may provoke a stress response [43].

### The impacts of chronic stress

Glucocorticoid release under challenging conditions is an adaptation to life in an unpredictable and threatening world [20]; individuals benefit from curtailing reproduction, altering behavioral patterns, and redirecting metabolic substrates to maximize glucose availability for action in response to genuine threats. Glucocorticoid levels alone are not directly or inversely correlated with fitness measures under all conditions [65], however, chronic adrenal activation has many known trade-offs that result in vulnerability to disease and death [22]. Unlike threats from predators, food shortages and inclement weather, noise typically does not directly threaten the survival of an individual or its offspring (though there may be exceptions, as discussed below). Therefore, the cost of chronic adrenal activation in response to noise pollution is unlikely to be outweighed by the benefits in most cases, and thus the net result may be adverse.

One important trade-off is the effect of corticosterone on immune response. Chickens infected with West Nile Virus (WNV) and administered corticosterone had increased oral shedding and lengthened duration of viremia compared to those without elevated cort [66]. For sage-grouse, which are highly susceptible to WNV [67], [68], reduced immune response due to elevated glucocorticoid levels could have a significant effect on survival in areas where they are exposed to WNV. Therefore, despite the adaptive nature of the stress response under natural conditions, elevated glucocorticoid levels due to human disturbance may have detrimental long-term impacts on welfare and survival of sage-grouse and other wildlife.

### Stress as an indicator of human impacts on sage-grouse

Measurement of FCMs may provide a non-invasive monitoring tool to assess the impact of human development (e.g. oil and gas drilling, wind farms, highways, off-road vehicle traffic) on stress levels of greater sage-grouse and other species. However comparisons between disturbed and undisturbed areas would need to account for differences in age, sex, and breeding condition of individuals sampled as well as for differences in the environmental conditions between sites in order to isolate stress as the likely cause of change [15], [18], [69]. We controlled for such differences by using an experimental presentation of noise that minimized effect on other habitat variables, limiting our collection to lekking birds, collecting only on days with limited female attendance and collecting samples from all leks within a short 2–3 day window. We did not find support for differences in FCM levels from samples collected in early versus late April within each season (∼20 days apart in a 2–3 month breeding season), and only limited evidence for an effect of location (Hudson vs. Riverton, ∼32 kilometers apart), suggesting that these temporal and spatial differences did not affect FCM levels in our study. However with a larger sample of leks or in another region or time period, it is possible that such differences might emerge.

### Conclusions

Taken together, results from Blickley et al. [43] and this study suggest that noise alone can cause greater sage-grouse to avoid otherwise suitable habitat and increase the stress responses of birds that remain in noisy areas. Thus, noise mitigation may be a fruitful conservation measure for this species of concern. In this study, we focused on the effects of noise from roads and drilling rigs in natural gas development areas; other natural gas development infrastructure, including compressor stations and generators, produces noise similar to drilling rigs, with the potential for similar effects on FCM levels. Likewise, other types of energy development produce noise similar in frequency, timing, and amplitude to the noise sources used here, including shale gas, coal-bed methane, oil, and geothermal development. The noise sources used in this study also share some characteristics with other anthropogenic noise sources that are increasing across the landscape, like wind turbines, off-road vehicles, highways and urban development; this suggests that the impacts on greater sage-grouse observed here may be widespread. More generally, populations of many species of birds [4], [70]–[74] and mammals [75]–[78] decline with proximity to noisy human activities, such as roads, urban and industrial developments. While further study is needed to determine whether chronic noise exposure contributes to the impacts of these human activities by activating the chronic stress response, this study adds to a growing body of evidence that such noise pollution is a threat to wildlife [1], [2], significantly increasing our estimates of the footprint of human development beyond the boundaries of visible disturbance.

## References

[1] Kight CR, Swaddle JP (2011) How and why environmental noise impacts animals: an integrative, mechanistic review. Ecology Letters 14: 1052–1061. 21806743 10.1111/j.1461-0248.2011.01664.x

[2] Barber JR, Crooks KR, Fristrup KM (2009) The costs of chronic noise exposure for terrestrial organisms. Trends in Ecology & Evolution 25: 180–189. 19762112 10.1016/j.tree.2009.08.002

[3] Blickley JL, Patricelli GL (2010) Impacts of Anthropogenic Noise on Wildlife: Research Priorities for the Development of Standards and Mitigation. Journal of International Wildlife Law and Policy 13: 274–292.

[4] Francis CD, Ortega CP, Cruz A (2009) Noise Pollution Changes Avian Communities and Species Interactions. Current Biology 19: 1415–1419. 19631542 10.1016/j.cub.2009.06.052

[5] Habib L, Bayne EM, Boutin S (2007) Chronic industrial noise affects pairing success and age structure of ovenbirds Seiurus aurocapilla. Journal of Applied Ecology 44: 176–184.

[6] Bayne E, Habib L, Boutin S (2008) Impacts of Chronic Anthropogenic Noise from Energy-Sector Activity on Abundance of Songbirds in the Boreal Forest. Conservation Biology 22: 1186–1193. 18616740 10.1111/j.1523-1739.2008.00973.x

[7] Hu Y, Cardoso GC (2009) Are bird species that vocalize at higher frequencies preadapted to inhabit noisy urban areas? Behavioral Ecology 20: 1268–1273.

[8] Slabbekoorn H, Ripmeester EAP (2008) Birdsong and anthropogenic noise: implications and applications for conservation. Molecular Ecology 17: 72–83. 17784917 10.1111/j.1365-294X.2007.03487.x

[9] Warren PS, Katti M, Ermann M, Brazel A (2006) Urban bioacoustics: it's not just noise. Animal Behaviour 71: 491–502.

[10] Patricelli GL, Blickley JL (2006) Overview: Avian communication in urban noise: the causes and consequences of vocal adjustment. The Auk 123: 639–649.

[11] Francis CD, Kleist NJ, Ortega CP, Cruz A (2012) Noise pollution alters ecological services: enhanced pollination and disrupted seed dispersal. Proceedings of the Royal Society B: Biological Sciences. 10.1098/rspb.2012.0230 PMC3367785 22438504

[12] Wikelski M, Cooke SJ (2006) Conservation physiology. Trends in Ecology & Evolution 21: 38–46. 16701468 10.1016/j.tree.2005.10.018

[13] Romero LM (2004) Physiological stress in ecology: lessons from biomedical research. Trends in Ecology & Evolution 19: 249–255. 16701264 10.1016/j.tree.2004.03.008

[14] Creel S, Fox JE, Hardy A, Sands J, Garrott B, et al (2002) Snowmobile Activity and Glucocorticoid Stress Responses in Wolves and Elk. Conservation Biology 16: 809–814.

[15] Wasser SK, Bevis K, King G, Hanson E (1997) Non-Invasive Physiological Measures of Disturbance in the Northern Spotted Owl. Conservation Biology 11: 1019–1022.

[16] Thiel D, Jenni-Eiermann S, Palme R (2005) Measuring corticosterone metabolites in droppings of capercaillies (*Tetrao urogallus*). Bird Hormones And Bird Migrations: Analyzing Hormones In Droppings And Egg Yolks And Assessing Adaptations In Long-Distance Migration. pp. 96–108. 10.1196/annals.1343.009 16055846

[17] Wasser SK, Bevis K, King G, Hanson E (1997) Noninvasive Physiological Measures of Disturbance in the Northern Spotted Owl. Conservation Biology 11: 1019–1022.

[18] Millspaugh JJ, Washburn BE (2004) Use of fecal glucocorticoid metabolite measures in conservation biology research: considerations for application and interpretation. General and Comparative Endocrinology 138: 189–199. 15364201 10.1016/j.ygcen.2004.07.002

[19] Lucas JR, Freeberg TM, Egbert J, Schwabl H (2006) Fecal corticosterone, body mass, and caching rates of Carolina chickadees (*Poecile carolinensis*) from disturbed and undisturbed sites. Hormones and Behavior 49: 634–643. 16458312 10.1016/j.yhbeh.2005.12.012 PMC1540716

[20] Wingfield JC (2005) The concept of allostasis: coping with a capricious environment. Journal of Mammalogy 86: 248–254.

[21] McEwen BS, Wingfield JC (2003) The concept of allostasis in biology and biomedicine. Hormones and Behavior 43: 2–15. 12614627 10.1016/s0018-506x(02)00024-7

[22] Sapolsky RM, Romero LM, Munck AU (2000) How do glucocorticoids influence stress responses? Integrating permissive, suppressive, stimulatory and preparative actions. Endocrine Reviews 21: 55–89. 10696570 10.1210/edrv.21.1.0389

[23] Wingfield JC, Sapolsky RM (2003) Reproduction and resistance to stress: when and how. J Neuroendocrinol 15: 711–724. 12834431 10.1046/j.1365-2826.2003.01033.x

[24] Opplinger A, Clobert J, Lecomte J, Lorenzon P, Boudjemadi K, et al (1998) Environmental stress increases the prevalence and intensity of blood parasite infection in the common lizard Lacerta vivipara. Ecology Letters 1: 129–138.

[25] Alario P, Gamallo A, Beato MJ, Trancho G (1987) Body weight gain, food intake and adrenal development in chronic noise stressed rats. Physiology & Behavior 40: 29–32. 3039551 10.1016/0031-9384(87)90181-8

[26] Barrett AM, Stockham MA (1963) The effect of housing conditions and simple experimental procedures upon the corticosterone level in the plasma of rats. Journal of Endocrinology 26: 97–105. 13969520 10.1677/joe.0.0260097

[27] Davis M, Walker DL, Lee Y (1997) Amygdala and bed nucleus of the stria terminalis: differential roles in fear and anxiety measured with the acoustic startle reflex. Philosophical Transactions of the Royal Society of London, B 352: 1675–1687. 10.1098/rstb.1997.0149 PMC1692102 9415919

[28] Atkinson H, Wood S, Kershaw Y, Bate E, Lightman S (2006) Diurnal Variation in the Responsiveness of the Hypothalamic-Pituitary-Adrenal Axis of the Male Rat to Noise Stress. Journal of neuroendocrinology 18: 526–533. 16774501 10.1111/j.1365-2826.2006.01444.x

[29] Thiel D, Jenni-Eiermann S, Palme R, Jenni L (2011) Winter tourism increases stress hormone levels in the Capercaillie *Tetrao urogallus* . Ibis 153: 122–133.

[30] Delaney DK, Grubb TG, Beier P, Pater LL, Reiser MH (1999) Effects of helicopter noise on Mexican spotted owls. Journal of Wildlife Mangement 63: 60–76.

[31] Tempel DJ, Gutiérrez RJ (2003) Fecal Corticosterone Levels in California Spotted Owls Exposed to Low-Intensity Chainsaw Sound. Wildlife Society Bulletin 31: 698–702.

[32] Babisch W (2003) Stress hormones in the research on cardiovascular effects of noise. Noise Health 5: 1–11. 12631430

[33] Ising H, Ising M (2002) Chronic cortisol increases in the first half of the night caused by road traffic noise. Noise Health 4: 13–21. 12537837

[34] Evans GW, Lercher P, Meis M, Ising H, Kofler WW (2001) Community noise exposure and stress in children. Journal Of The Acoustical Society Of America 109: 1023–1027. 11303916 10.1121/1.1340642

[35] Evans GW, Bullinger M, Hygge S (1998) Chronic Noise Exposure and Physiological Response: A Prospective Study of Children Living under Environmental Stress. Psychological Science 9: 75–77.

[36] Department of the Interior (2010) Endangered and Threatened Wildlife and Plants; 12-Month Findings for Petitions to List the Greater Sage-Grouse (*Centrocercus urophasianus*) as Threatened or Endangered. Federal Register 75: 13910–14014.

[37] Connelly JW, Knick ST, Schroeder MA, Stiver SJ (2004) Conservation Assessment of Greater Sage-grouse and Sagebrush Habitats. Cheyenne, WY: West. Assn. Fish and Wildlife Agencies. 610 p.

[38] Holloran MJ (2005) Greater Sage-Grouse (*Centrocercuc urophasianus*) Population Response to Natural Gas Field Development in Western Wyoming [Dissertation]. Laramie: University of Wyoming. 114 p.

[39] Walker BL, Naugle DE, Doherty KE (2007) Greater Sage-Grouse Population Response to Energy Development and Habitat Loss. Journal of Wildlife Management 71: 2644–2654.

[40] Holloran M, Kaiser R, Hubert W (2010) Yearling greater sage-grouse response to energy development in Wyoming. Journal of Wildlife Management 74: 65–72.

[41] Naugle DE, Doherty KE, Walker BE, Holloran MJ, Copeland HJ (2011) Energy development and Greater Sage-Grouse. In: Knick ST, Connelly JW, editors. Greater Sage-Grouse: ecology and conservation of a landscape species and its habitats. Berkeley, CA.: University of California Press.

[42] Blickley JL, Patricelli GL (2012) Potential acoustic masking of greater sage-grouse display components by chronic industrial noise. Ornithological Monographs 74: 23–35.

[43] Blickley JL, Blackwood D, Patricelli GL (2012) Experimental Evidence for the Effects of Chronic Anthropogenic Noise on Abundance of Greater Sage-Grouse at Leks. Conservation Biology 26: 461–471. 22594595 10.1111/j.1523-1739.2012.01840.x

[44] Vehrencamp S, Bradbury J, Gibson R (1989) The energetic cost of display in male sage grouse. Animal Behaviour 38: 885–896.

[45] Jankowski MD (2007) The influence of habitat disturbance and synergized resmethrin on avian immunocompetence: The University of Wisconsin, Madison.

[46] Jankowski MD, Wittwer DJ, Heisey DM, Franson JC, Hofmeister EK (2009) The Adrenocortical Response of Greater Sage Grouse (*Centrocercus urophasianus*) to Capture, ACTH Injection, and Confinement, as Measured in Fecal Samples. Physiological and Biochemical Zoology 82: 190–201. 19199814 10.1086/596513 PMC2666624

[47] Wasser SK, Hunt KE, Brown JL, Cooper K, Crockett CM, et al (2000) A Generalized Fecal Glucocorticoid Assay for Use in a Diverse Array of Nondomestic Mammalian and Avian Species. General and Comparative Endocrinology 120: 260–275. 11121291 10.1006/gcen.2000.7557

[48] Burnham KP, Anderson DR (2002) Model selection and multimodel inference: a practical information-theoretic approach: Springer Verlag.

[49] Weiss JM (1970) Somatic Effects of Predictable and Unpredictable Shock. Psychosomatic Medicine 32: 397–408. 5535207 10.1097/00006842-197007000-00008

[50] Wingfield JC, Maney DL, Breuner CW, Jacobs JD, Sharon L, et al (1998) Ecological Bases of Hormone-Behavior Interactions: The “Emergency Life History Stage”. American Zoologist 38: 191–206.

[51] Goymann W, Wingfield JC (2004) Allostatic load, social status and stress hormones: the costs of social status matter. Animal Behaviour 67: 591–602.

[52] Angelier F, Shaffer SA, Weimerskirch H, Chastel O (2006) Effect of age, breeding experience and senescence on corticosterone and prolactin levels in a long-lived seabird: The wandering albatross. General and Comparative Endocrinology 149: 1–9. 16750533 10.1016/j.ygcen.2006.04.006

[53] Angelier F, Moe B, Weimerskirch H, Chastel O (2007) Age-specific reproductive success in a long-lived bird: do older parents resist stress better? Journal of Animal Ecology 76: 1181–1191. 17922714 10.1111/j.1365-2656.2007.01295.x

[54] Schwabl H (1999) Developmental Changes and Among-Sibling Variation of Corticosterone Levels in an Altricial Avian Species. General and Comparative Endocrinology 116: 403–408. 10603278 10.1006/gcen.1999.7379

[55] Wada H, Hahn TP, Breuner CW (2007) Development of stress reactivity in white-crowned sparrow nestlings: Total corticosterone response increases with age, while free corticosterone response remains low. General and Comparative Endocrinology 150: 405–413. 17150217 10.1016/j.ygcen.2006.10.002

[56] Freeman BM (1982) Stress non-responsiveness in the newly-hatched fowl. Comparative Biochemistry and Physiology Part A: Physiology 72: 251–253.

[57] Dickens MJ, Romero LM (2010) Stress Responsiveness Decreases With Age in Precocial, Juvenile Chukar. The Wilson Journal of Ornithology 122: 762–766.

[58] Rubenstein DR (2007) Stress hormones and sociality: integrating social and environmental stressors. Proceedings of the Royal Society B: Biological Sciences 274: 967–975. 17251100 10.1098/rspb.2006.0051 PMC2141667

[59] Brumm H, Slabbekoorn H (2005) Acoustic Communication in Noise. Advances in the Study of Behavior 35: 151–209.

[60] Mockford EJ, Marshall RC (2009) Effects of urban noise on song and response behaviour in great tits. Proceedings of the Royal Society B: Biological Sciences 276: 2979–2985. 19493902 10.1098/rspb.2009.0586 PMC2817215

[61] Swaddle JP, Page LC (2007) High levels of environmental noise erode pair preferences in zebra finches: implications for noise pollution. Animal Behaviour 74: 363–368.

[62] Rabin LA, Coss RG, Owings DH (2006) The effects of wind turbines on antipredator behavior in California ground squirrels (Spermophilus beecheyi). Biological Conservation 131: 410–420.

[63] Quinn L, Whittingham J, Butler J, Cresswell W (2006) Noise, predation risk compensation and vigilance in the chaffinch Fringilla coelebs. Journal of Avian Biology 37: 601–608.

[64] Dooling RJ, Popper AN (2007) The Effects of Highway Noise on Birds. Sacramento, CA: The California Department of Transportation Division of Environmental Analysis. 74 p.

[65] Bonier F, Martin PR, Moore IT, Wingfield JC (2009) Do baseline glucocorticoids predict fitness? Trends in Ecology & Evolution 24: 634–642. 19679371 10.1016/j.tree.2009.04.013

[66] Jankowski MD, Franson JC, Möstl E, Porter WP, Hofmeister EK (2010) Testing independent and interactive effects of corticosterone and synergized resmethrin on the immune response to West Nile virus in chickens. Toxicology 269: 81–88. 20096745 10.1016/j.tox.2010.01.010 PMC2861826

[67] Naugle DE, Aldridge CL, Walker BL, Cornish TE, Moynahan BJ, et al (2004) West Nile virus: Pending crisis for Greater Sage-Grouse. Ecology Letters 7: 704–713.

[68] Walker BE, Naugle DE (2011) West Nile Virus ecology in sagebrush habitat and impacts on Greater Sage-Grouse populations. In: Knick ST, Connelly JW, editors. Greater Sage-Grouse: ecology and conservation of a landscape species and its habitats. Berkeley, CA.: University of California Press.

[69] Sheriff MJ, Wheeler H, Donker SA, Krebs CJ, Palme R, et al (2012) Mountain-top and valley-bottom experiences: the stress axis as an integrator of environmental variability in arctic ground squirrel populations. Journal of Zoology 287: 65–75.

[70] Kuitunen M, Rossi E, Stenroos A (1998) Do Highways Influence Density of Land Birds? Environmental Management 22: 297–302. 9465138 10.1007/s002679900105

[71] van der Zande AN, ter Keurs WJ, van der Weijden WJ (1980) The impact of roads on the densities of four bird species in an open field habitat–evidence of a long-distance effect. Biological Conservation 18: 299–321.

[72] Rheindt FE (2003) The impact of roads on birds: Does song frequency play a role in determining susceptibility to noise pollution? J für Ornithologie 144: 295–306.

[73] Ingelfinger F, Anderson S (2004) Passerine response to roads associated with natural gas extraction in a sagebrush steppe habitat. Western North American Naturalist 64: 385–395.

[74] Peris SJ, Pescador M (2004) Effects of traffic noise on paserine populations in Mediterranean wooded pastures. Applied Acoustics 65: 357–366.

[75] Singer FJ (1978) Behavior of Mountain Goats in Relation to US Highway 2, Glacier National Park, Montana. The Journal of Wildlife Management 42: 591–597.

[76] Rost GR, Bailey JA (1979) Distribution of Mule Deer and Elk in Relation to Roads. The Journal of Wildlife Management 43: 634–641.

[77] Forman RTT, Deblinger RD (2000) The Ecological Road-Effect Zone of a Massachusetts (U. S. A.) Suburban Highway. Conservation Biology 14: 36–46.

[78] Sawyer H, Kauffman M, Nielson R (2009) Influence of well pad activity on winter habitat selection patterns of mule deer. Journal of Wildlife Management 73: 1052–1061.

